# York County Hospital Inquiry

**Published:** 1913-06-07

**Authors:** 


### YORK COUNTY HOSPITAL INQUIRY.

### REPORT OF SIR E. COOPER PERRY, M.D., COMMISSIONER, AND MR. BASIL WATSON, LEGAL ASSESSOR.

THE full text of the report is as follows :—

1. In a letter, dated March 28, 1913, addressed to me by Mr. Frederick Neden, secretary and manager of the York County Hospital, I was informed that it was proposed to appoint a gentleman " with knowledge of hospital administration and free from local influence " to conduct an inquiry into the charges lately made against the hospital. The nomination of the Commissioner was to be made by Sir Rickman Godlee, Bart., President of the Royal College of Surgeons of England, and it was left to the gentleman appointed " to decide as to the method of conducting the inquiry."

2. In reply to this letter I stated that " having now read the allegations made by your late residents in the copy of THE HOSPITAL newspaper sent to me, I was satisfied that the inquiry was not one that I should in any case be prepared to make without the assistance of a legal coadjutor." On April 3 I learnt that the House Committee, not wishing to incur any expense that could be avoided, would have preferred me to undertake the inquiry myself. After hearing my reply to this suggestion, the Committee, on April 5, acceded to my desire for legal assistance, and on my nomination Mr. Basil Watson, barrister-at-law, of 1 Tanfield Court, Temple, was appointed legal assessor. The Committee arranged to provide for shorthand notes and a transcription, and the complete transcript of the shorthand notes (about 76,000 words) reached me on Monday, the 5th instant.

3. The inquiry was held in the Board Room of the County Hospital, York, on Friday and Saturday, April 18 and 19, occupying thirteen hours in all, and our formal reference was as follows :—

" That, with the assistance of Mr. Basil Watson, as legal assessor, I should conduct an inquiry into the charges made against this hospital published in THE HOSPITAL newspaper of March 15, and should furnish the Committee with a report on the result of my investigations." (Secretary's letter, April 8, 1913.)

4. By a letter from the Secretary of April 14 I learnt that it had already been settled to publish my report, and it was left to us to decide as to the presence of the Press. In a letter to the Secretary on the following day we stated that we were of opinion " that admission should not be refused to representatives of the Press who might desire to attend our inquiry, and that we should be obliged if arrangements might be made accordingly."

5. As above-mentioned, the Governors of the hospital were desirous that I should assume full responsibility for the inquiry, " forming my own opinion and reporting," and this responsibility I assume ; but I think I am fortunate in having secured for my conclusions the concurrence of my assessor, who, whilst not an expert in hospital administration, is a gentleman of varied legal experience, approaching the matters under consideration from the point of view of the general public ; nor can I but believe that the report thus signed by both of us will carry more weight than if signed independently by myself.

6. At the inquiry the hospital was represented by Mr. Alfred Procter, solicitor to the hospital, and Drs. Macqueen and Shepherd were represented by their counsel, Mr. F. S. Arnold Baker, instructed by Messrs. Baker and Nairne, solicitors, of 3 Crosby Square, Bishopsgate, E.C.

7. It may perhaps here be convenient to record that Mr. J. G. Macqueen, M.B., Ch.B., Edin., commenced his duties as house physician at the York County Hospital on May 26, 1912, and succeeded as house surgeon on August 13; that Mr. W. Moir Shepherd, M.B., Ch.B., Edin., entered upon his appointment as house physician on September 9, and that both gentlemen retired on January 23, 1913. Dr. Macqueen graduated in July 1911, and had had previous experience at the Leicester Royal Infirmary; Dr. Shepherd graduated in July 1912, and had held no resident appointment elsewhere.

8. In investigating the various charges made by the late residents against the hospital we dealt with them, as far as practicable, in the order in which they appeared in the columns of THE HOSPITAL newspaper of March 15, 1913; and we propose to follow the same order in reporting upon them.

### I.

### Alleged Ill-Treatment of a Child.

The statement of the residents with regard to this case on page 645A of THE HOSPITAL is that on October 14, 1912 (October 13 is the actual date of the letter) they reported to this effect : " that a child in the children's ward was discovered by us with suspicious marks upon it, which we attributed to violence. Inquiries from the matron and nurses brought no satisfactory explanation of the marks."

This statement is thus amplified by Dr. Shepherd in a letter to the Medical Board of the above-mentioned date (October 13, 1912) :—

" I wish to call your attention to the following *re* Jimmie M., at present a patient in Victoria Ward. The above child is now convalescent from an empyema of the right side, and almost fit to go home. This morning (Sunday, October 13) I visited the ward and asked that the child's dressing be removed, as I wished to examine him, not having done so for some time, as everything was going on quite well, and sister reported the sinus as almost closed. I found everything as reported, but noticed on the left side of chest, and extending towards mid-axillary line, what appeared to me " marks " which might well have been bruises. As regards their age, I should feel inclined to state from " five to seven days " old. As the child appeared to be very frightened, and not what he had been on a previous occasion, I sought to make further inquiries from the nurse in charge, the sister being away for the week-end. Nurse was confused and did not seem to like my inquiries. She said she knew about it, but would rather not say at present. I had Dr. Macqueen sent for; he, too, examined the child, and was present when I questioned the nurse. I telephoned for Dr. Gayner, and said I should like him to see his case, but meantime thought I might as well see the matron. For some reason or other, perhaps best explained by herself, the matron was inclined to argue, and make various statements, amongst others, that I must have had information from some source, and, furthermore, she thought it not proper to look into such things when sister was away, saying it was underhand; moreover, she deliberately stated that I must have been making inquiries *re* conduct of sister in the ward, otherwise I should not have noticed anything the matter with the child."

The letter concludes with a request to the Medical Board to have the matter "fully investigated."

From notes supplied by Dr. Gayner, not taken on the spot, but written as soon as he returned home from the hospital, it appears that he was called on Sunday morning, at 10.30, per telephone, by Dr. Shepherd, and he came down to the hospital and examined the "marks" on the left side of the chest wall. He found the "mark" was not so obvious that he would have discovered its presence if his attention had not been directed to the question. It was less than half an inch across, and extended from the mid-axillary line upwards and backwards.

It appears from Dr. Evelyn's evidence that he also was shown the "marks," and, on looking at the child's back, said: "I am sorry, but I do not see anything at all on the child. Where are the marks? And a small mark was pointed out to me on the back of the left side—a small superficial mark due very probably to a wreathing of the bandage round the child's body. That was all I could find or see." And Dr. Evelyn contends that the mark that was shown to him by Dr. Shepherd as the result of the blow "was not the result of a blow at all."

Dr. Gayner repeated before us at our inquiry the statements above made in his notes, with the addition that it was abundantly shown at the subsequent inquiry by the sister and nurse concerned that the smack was given on the right side, the importance of this discrepancy being in Dr. Gayner's view that it affects the value of the evidence both of Dr. Shepherd and Dr. Macqueen, who say they saw marks of injury on the left side. Mr. Procter attached importance to this question as having a general bearing on the whole of their attitude in the charges they made.

The nurse, who had seen the child struck by the sister, informed us that he had been struck more than once—in fact, several times—and, as regards the matron's allegation that Dr. Shepherd's information was derived from the nurse, her evidence is distinct that no inquiries had been made of her by Dr. Shepherd whether anything had happened to the child before he had actually discovered the marks by his own observation.

Though we had it in evidence from the matron that she, too, was unable to see the alleged marks, nevertheless we have formed the opinion that the residents did actually see marks upon the child, which were the results of the ill-treatment he received from the sister.

In response to Dr. Shepherd's letter (13th) the Medical Board lost no time in investigating the circumstances, and we had before us the minutes of special meetings held by them in the morning and evening of October 16, when evidence was given by the residents, the matron, the sister of the Children's (Victoria) Ward, the nurse above mentioned, and another nurse.

The sister of the ward admitted that on October 6 she had struck a child in Victoria Ward severely, and had not reported the occurrence. On the 14th Dr. Gayner reports the matron as stating that the nurse had informed her that she saw the sister strike the patient a week ago, and had seen the bruises during the past week.

Under these circumstances the Medical Board decided to recommend the matron to accept the resignation of the sister, and to report the same to the House Committee; and it appears that the sister ceased to be on duty from October 15, and quitted the service of the hospital on October 22. She came to the hospital to take charge of the children's ward on May 26, 1911.

We report as to this allegation that the result of the investigation undertaken by the Medical Board justified the residents in making this complaint, and that no undue delay occurred in taking effective measures when once the circumstances had been brought to the cognisance of the Medical Board and House Committee.

II.

### Mischarting in the Children's Ward.

The second allegation (The Hospital, page 645a) is that "in the same ward the temperature of a case had been wilfully mischarted . . . Complaint had been made by us to the matron previously about similar occurrences in the same ward, and no notice had been taken of it. Also that in many cases there were no records of pulse or respirations, even in a case of osteomyelitis (in this case there was no record for over seven weeks)."

This complaint is set out at greater length in a letter from Dr. Macqueen to the Medical Board of October 14, 1912, in which the resident states that he wishes to call the attention of the Board to the case of Margaret M., in Victoria Ward, one of Dr. Micklethwaite's cases.

"One evening, as I was doing my round, I noticed this child's chart, and found that on October 1 her temperature had been charted as 101.6. This had been scratched out and recharted as 100.6. On asking the staff nurse why such a mess was made of the chart, I was informed that she on that particular day had taken the temperature herself, and that it was 101.6; but that the sister of the ward, without herself taking the temperature, had erased this and charted it as 100.6."

"I have on a previous occasion laid a complaint before the matron of similar occurrences in the same ward, and, as it has again happened, I think it is time the matter was looked into by the Medical Board. At the same time may I point out that on many of the charts in the same ward there will be found no record of pulse or respirations except on the day of admission, even though the case may have been in the ward for several weeks? One case in particular in which I think the pulse, etc., is of importance—viz. a case of osteomyelitis of the arm—which has not had the pulse or respiration recorded for seven weeks. In this child I had to open another abscess yesterday."

These charges by the residents we report to be fully justified, having regard to the fact that, before the Medical Board on October 16, the same sister admitted that "it was not her custom to record high temperatures unless they were such as from the condition of the child she would expect; her custom being to have the temperatures taken again after some lapse of time, and to record only the lower temperatures."

III.

### Alleged Inadequacy of the Nursing Staff.

The allegations of the residents under this head (The Hospital, p. 645 a and b) are as follows:—

"That in our opinion the night staff of the whole hospital was inadequate for the treatment of the patients; besides there being no emergency night staff to take theatre or special duty if required."

In support of this charge two cases were cited:—

(1) "In one ward there was a surgical case which required a great deal of attention. While the ward nurse was attending to this case, a patient in the other division of the ward got up, slipped on the floor, and broke his leg."

(2) "In another ward there was a medical patient who twice fell out of bed. There was one nurse who was doing special duty between these two cases, and in each the 'part duty' resulted in the above."

The facts with regard to the first of these cases appear to be well ascertained. The patient was a youth, seventeen or eighteen years of age, admitted for traumatic synovitis of the knee. He was well enough to be allowed to get up, had no mental symptoms, and was just about to be discharged from the hospital cured. He got out of bed for some purpose unknown, slipped, and broke his thigh. Much as the accident is to be regretted, it is difficult to see how, short of providing each patient with a special attendant, such a mishap could be effectively prevented.

The other cases were of a different character. One was a patient upon whom a splenectomy had been performed, followed by septic complications. There is no question that a special nurse ought to have been, and in fact was, provided for him. The other case was a girl in a ward on another floor, suffering from a severe form of St. Vitus's Dance (Chorea insaniens). She was under the care of Dr. Gayner, and it appears that when he saw her in the morning before the night on which she fell out of bed he was asked whether he would order a "special" nurse, and he said No. In the evening the patient got much worse, and a "special" became necessary. At this point there is a conflict of testimony as to whether the house surgeon, under whose care the splenectomy was, and the house physician, under whose care the chorea case was, agreed that one "special" nurse should be shared between the two patients. As the necessity for the "special" only became urgent about midnight, the question was not referred to the matron, but to the night sister, and her evidence is that the house physician and the house surgeon consented that one "special" nurse should serve two cases. She states that the residents were both in the ward at the same time, and they agreed that the "special" should go between the two floors, and moreover that "they said it quite nicely, too." This arrangement is flatly denied by the residents, and it is a question between them and the night sister.

We report that in this matter the evidence of the residents is, in our judgment, more accurate than that of the night sister. In any case, according to the matron's statement, the night sister had a second nurse available as a "special" had she seen fit to employ her in that capacity. We do not, therefore, accept these particular instances as proof of the inadequacy of the nursing staff, but the question is further considered later in our report under XIX.

### IV.

### Complaints Against the Matron.

In a letter (undated, but referred to in The Hospital as of December 27, 1912), the residents complain that the matron, when the condition of affairs above recorded in the children's ward was reported to her, "instead of taking such a report from a resident as a matron should, wished to argue the matter, and deliberately shut her eyes to the delinquencies involved. The sister was afterwards caught red-handed, and on this being reported to the matron the latter was exceptionally (or, as it appears in The Hospital, p. 645b, exceedingly) insolent."

In reference to this charge we have found it very difficult to define what the proper attitude of a matron should be when she receives a resident's report, nor are any actual words quoted to us as evidence of her "insolence" to the house officers.

What, however, seems to have been the case is that, when the matron received these representations from the residents as to the irregularities which they found existing in the children's ward, instead of instituting an immediate inquiry on her own account, she adopted the view that "the sister was a very capable woman, who had been well recommended to her, and she did not see how such things could be." As a matter of fact the event has shown that she was wrong, and that the residents were right in their suspicions as regards this particular ward.

Generally we judge that the matron kept the residents at arm's length, and the gravamen of their charge against her is that when complaints were brought by them to her notice she did not investigate them, so far as they knew, or at any rate they did not know what she did; and that out of courtesy at least she should have told them what the result of her investigation was. Thus put, we think that there was some ground for their dissatisfaction, but, on the other hand, we have not heard the matron's side of the story. Without endeavouring to apportion the blame between the two parties, we can have no doubt that the interests of the hospital suffered from the strained relations which existed between the matron and the residents.

### V.

### Children Tied Down in Bed.

At this point it may be convenient to take the charge which appears as 17 on p. 647 of The Hospital. It is as follows:—

"When we first went to the hospital we found that it was customary to tie the children down in bed, no matter what the case might be. The method of tying was by bandage round the shoulders, wrists, and ankles."

Dr. Macqueen explained that this charge also related to the children's ward, and to the same sister, the circumstances of whose resignation have already been recorded under I.

It appears from Dr. Macqueen's evidence that on the first day of his arrival in the hospital he discovered more than six children tied down, whilst Dr. Shepherd subsequently found two children tied down, and in each case, without reporting the facts to the honorary staff, they cut the bandages, thus effectively indicating their condemnation of the practice.

We had it in evidence from Dr. Evelyn that the custom had been in vogue of "tying down" children ever since he had been connected with the hospital, and he demonstrated to us a method, which he regarded as unobjectionable, of tying down by means of a loop passed over the child's body with armlets attached, but leaving the hands and feet free. He also urged that in the case of children suffering from widespread eczema it might occasionally be desirable that they should be restrained from scratching themselves by confining both the hands and feet.

The evidence, however, of the residents is to the effect that they never saw what may be described as the approved form of restraint employed at all. What they found was a practice of tying the child by bandage round shoulders, hands, and feet, and from what we know of the condition of things then existing in the children's ward we accept their evidence on this matter.

Probably they said nothing to the honorary staff because they had heard of the custom mentioned by Dr. Evelyn without troubling accurately to inform themselves how the restraint should be applied, and, as they did not approve of what they actually saw, they solved the difficulty in the way which seemed simplest to them by cutting the bandages and setting the children free.

Dr. Macqueen states that he informed the matron of his objections to the tying down of the children about the beginning of September. The matron says that "the matter of tying down the children was not one of the complaints made to me." It seems to us likely that it may have been actually mentioned to her, but that, knowing it to be the custom of the hospital, and having confidence in the sister of the ward, she did not investigate the matter so far as to discover whether the tying down was carried out by means of the apparatus recognised and approved by the honorary staff or by the crude method condemned by the residents.

VI.

### Patient Removed to the Mortuary.

The first account of this case is contained in the following letter (September 27, 1912) from Dr. Shepherd to Dr. Gayner, the honorary physician, under whose care the patient was :—

"*Re* C., who died Sunday, the 22nd, at 4.45 P.M. in the above (York County) Hospital. C. suffered from chronic nephritis, and for some days previous to his death had manifested slight symptoms of uræmic coma. On Sunday, September 22, at 7.50 A.M., Nurse H. came to my bedroom, and said C. did not look so well, and that his pulse was failing. Knowing his condition, I ordered him a hot pack. At 8.5 A.M. Sister H. came to me with a similar report, and I told her I had ordered a hot pack, after which I heard nothing further until 9 A.M., when the porter informed me that C. was dead and had been removed to the mortuary.

"At 11.45 I went over to the mortuary to have a look at the body, which was laid out in the usual manner. I was struck by the warmth and general appearance of the body, and led to make further examination, which fully convinced me that C., though in deep coma, still lived.

"I immediately summoned Dr. Macqueen, who at once recognised that life was not extinct.

"Together we removed C. to the isolation ward, where, however, he did not improve, and finally expired at 4.45 P.M.

"The wife had already been informed of his death, but I had little difficulty in explaining to her the real situation, and as she did not, to my knowledge, know he had been in the mortuary, she expressed herself as quite satisfied, and a death certificate was duly given to her."

In the joint letter of the residents dated January 11, 1913, the statement, which is exactly as printed in THE HOSPITAL, appears as follows :—

"On September 22, 1912, a patient named C. was discovered about 11.30 A.M. by the house physician in the mortuary whilst still alive. A ticket pinned upon the shroud had written upon it that the patient died at 7.30 A.M., and yet at 7.50 A.M. the house physician was asked concerning the case, and gave his orders there anent; which orders, he afterwards found, were not carried out; nor was he informed of the 'death' until after the patient had been removed to the mortuary.

"Upon the discovery of this fact we decided to remove the patient to the isolation ward, and informed the matron of our decision, but were met with the astounding reply, 'What advantage is there in having him removed now?'

"The patient ultimately died in the isolation ward at 4.45 P.M."

The facts of the case, as they came out in evidence, were fairly simple. The patient was suffering from chronic Bright's disease, and had shown signs of uræmia. On the morning of Sunday, September 22, 1912, about 7.10, he was washed by the day nurse, and at 7.40 spoke to the night sister (Sister H.). At 7.50 the day nurse, turning round from making the beds of the other patients, noticed that C. was cyanosed and becoming convulsed. An attempt was then made to find the night sister; as she was not immediately available, the night nurse (Nurse H.) was sent, about five minutes to eight, to Dr. Shepherd's room to report the circumstances and ask for instructions.

She returned to the ward with an order from Dr. Shepherd that a hot pack should be administered. Here she met the night sister, who went to the patient's bed, and, finding him as she thought sinking very quickly, went herself about five minutes past eight to Dr. Shepherd's bedroom, thinking, as she says, that something might be done whilst they were getting ready the hot pack.

The hot pack was not given, because whilst preparations were being made the patient appeared to die. The day nurse says that she "could feel his pulse, but that it was just a flicker, and afterwards it was nothing." The fact of the patient's death seems to have been accepted by the night sister, who went off duty at 8.15, by the night nurse, and by the ward sister who took the night report from the night nurse, and herself only saw the body laid on the bed.

It is not perhaps surprising that the day sister did not herself examine the patient, since she somehow got from the night nurse the impression he had actually died at half-past seven, and filled up the mortuary ticket to that effect.

The day nurse proceeded with the work of laying out the body, which was completed by nine o'clock, or a little after, and between nine and half-past, according to the evidence of the sister and the mortuary porter, the body was removed upon the sister's order to the mortuary.

Between 11.30 and 11.45 Dr. Shepherd, being about to do some work there, and desiring also to see the corpse before he signed the death certificate, went to the mortuary, and came to the conclusion that the man was still alive, finding that his heart was beating and that "he had a pulse at the wrist, although extremely thready."

Dr. Shepherd immediately called in Dr. Macqueen, who, according to his evidence, was in the mortuary between 11.30 and 11.45, and he found that the pulse could be felt very slightly in the radial artery. To make assurance doubly sure, and also, as he says, by way of treatment for his uræmic condition, Dr. Macqueen severed the patient's radial artery, from which, according to Dr. Shepherd, there was "a continuous spurt" of blood, and, at the assistant-matron's subsequent visit to the mortuary, there was blood on the floor, amounting in her calculation to one or two ounces.

Steps were then taken by the residents themselves to move the body into the isolation block, and instructions were given for the room to be got ready.

According to Dr. Shepherd, it was only twenty minutes to half an hour before they succeeded in moving the body. Pressed further upon this point, Dr. Shepherd places the time between twelve and one o'clock and possibly one o'clock, but asserts that at any rate the removal was effected before luncheon, the hour of which on Sundays is one to a quarter-past one o'clock, and Dr. Macqueen confirmed the statement that the body was moved before luncheon—before one o'clock. However this may be, when the body was transferred to the isolation block, though various measures were adopted, the patient gave no signs of returning vitality, and was finally judged to have expired at 4.45 P.M. The patient throughout was comatose and profoundly unconscious.

Exception was taken on the part of the hospital to the conduct of Dr. Shepherd in not himself visiting the ward when summoned by the night nurse, but, on the other hand, Dr. Shepherd's evidence is that between two and three o'clock in the morning of the Sunday on which the patient died he had already seen him, and that it was, therefore, sufficient for him to order a hot pack, and to wait to be informed of the result of the treatment, nor, however unfortunate it may be in the light of subsequent events that he did not go to the ward, can we judge him to be guilty of dereliction of duty in this matter.

The night sister gives a vivid account of her interview with Dr. Shepherd, alleging that, in response to her request that something might be done to keep the patient alive until his relatives could be communicated with, Dr. Shepherd replied "He is dead," and she adds, "I wondered at the time how he got the information that he was dead." We are bound to say that we do not accept this story, though supported to some extent by the matron's evidence, but it seems quite probable that Dr. Shepherd may have said "He is as good as dead," knowing that he was in uræmic coma. Nor do we think that Dr. Shepherd subsequently suggested to her that she should say nothing about the matter, when Dr. Gayner was holding his inquiry.

A more important point arises with reference to the hour at which Collier was removed from the mortuary to the isolation block. The residents are jointly pledged to the statement that it was before luncheon. On the other hand the matron's evidence is that some little time after one o'clock Dr. Macqueen came to see her, and informed her of the fact that the man was not dead, that she was naturally much surprised and distressed, and that she asked at once whether he was conscious. She then set about making arrangements for getting the isolation room ready, for which mattresses, bedding, etc., had to be sought. At a quarter to two o'clock, the necessary arrangements having by that time been completed, she came straight from the isolation block to the doctors' dining-room, where she told them that the ward was ready for the reception of the patient. The exact times of these proceedings are fixed in her mind by the fact that her Sunday luncheon is over by one o'clock, that she had been in her room about ten minutes before Dr. Macqueen's arrival, and that she was due to play the harmonium in the hospital chapel at a quarter to two o'clock, and, as a matter of fact, she was a little late, for she found the chaplain was waiting for her. If her statement is correct, the residents must have left the body in the mortuary whilst they themselves were having luncheon.

The evidence of the assistant matron is that at twenty minutes to one o'clock, while the sisters were at dinner, Dr. Shepherd came to her room to report that C. was still alive in the mortuary, that Dr. Macqueen had severed an artery, that it was still running, and "would I give him some dressings." Accordingly she went to the casualty room, got the dressings, and she and Dr. Shepherd proceeded to the mortuary.

At her suggestion it was arranged to take the man to the isolation room, and, when the arm had been stitched and bandaged by Dr. Macqueen, she returned with Dr. Shepherd to the casualty room, and, meeting Sister C. about 1.5 o'clock going out from dinner, and having herself to take the nurses' dinner, she gave instructions for Sister C. to get the mattresses, etc., for the isolation room. At half-past one she came out from the nurses' dinner, went to the store room to get the stores out, which took about a quarter of an hour, and proceeded to the mortuary between twenty minutes and a quarter to two, where C. was still lying. Wondering why, if the residents considered him alive, he was left there, she went into the isolation room, and found Sister C. waiting for him. As he had still not been transferred, she went after the doctors, inquired in the hall where they were, went to their dining-room, and found that they were having luncheon, and had not finished. It was then about ten minutes to two, and, as C.'s wife was expected at two o'clock, the assistant matron and the residents went all three to the mortuary, and removed him to the isolation ward. At that time Dr. Shepherd stated that he could still hear a faint flutter with a stethoscope over the patient's heart, but much less than when he examined him in the mortuary.

The evidence of Sister C. is to the effect that at twenty minutes to two she took a mattress to isolation ward, and that the patient had not at that time been removed from the mortuary.

We are bound to say that this cumulative evidence, which was quite unshaken by Mr. Baker's cross-examination, has convinced us that the residents left C. in the mortuary after their first discovery that he was still alive, and did not actually remove him until the assistant matron interrupted their luncheon.

There remains a further charge to be considered against the matron in this painful affair, viz.: that when the residents decided to remove the patient to the isolation ward, and informed the matron of their decision, they were "met with the astounding reply, 'What advantage is there in having him removed now?'"

As regards this remark, the matron states that she has no recollection whatever of making it. She has, however, a recollection that Dr. Macqueen said to her that the man was practically dead, but not legally dead, and that he was not conscious. Dr. Macqueen denies using these words, and very likely she is mistaken in attributing them to him, since his colleague admits having himself said that the man was virtually dead, not legally. Further, the matron's remark, he thinks, though he is not certain, followed on these expressions of his own, and this admission puts a somewhat different complexion upon the charge of "callous ineptitude" levelled against her in the residents' letter of December 27 to the chairman of the House Committee.

Nevertheless, however regarded, the incident, with all its details, is gruesome and shocking to the public mind, and it is satisfactory to know that, following upon an inquiry held on October 1 by Dr. Gayner, on behalf of the Medical Board, the House Committee, sixteen days after the mishap, sanctioned a new bye-law to the effect that "The resident officers shall not give a death certificate in respect of the death of an in-patient without an inspection of the body."

By a further resolution of the House Committee of January 14, 1913, when the case was first officially reported to them, the present rule runs as follows:—

"The resident officer shall not give a death certificate in respect of the body of any patient without an inspection of the body before its removal from the ward, and such removal shall not take place until an interval of two hours has elapsed since the death occurred."

The Medical Board has further recommended:—

(a) That, in the event of a death occurring in a ward, that death shall be entered by a sister who has inspected the body in the report book, and that the final stage of laying-out a body be not commenced until one hour after the entry of the death in the report book.

(b) In cases in which it is inadvisable to carry this out, it will be notified to the honorary officer who has had charge of the case in a report by the ward sister, countersigned by the matron.

The allegation (15) on page 647 of The Hospital that it seemed to be customary to lay out bodies and remove them to the mortuary as soon as possible was explained by Mr. Baker as referring only to this case, and no evidence was afforded that there was any general custom to that effect.

VII.

### Failure to Report a Burn on a Child.

The residents' statement in The Hospital (p. 646b) is that:—"On July 6, 1912, at 12.5 a.m., the matron reported to the house physician (present house surgeon) about a child in Victoria Ward having a bedsore. This had been present for twelve days, and had been treated without the knowledge of the resident. Arrangements had been made for the child to go out that day."

In the residents' letter of January 11, 1913, the last sentence runs as follows:—

"Arrangements had been made for the child to go out that day, and evidently this was the only reason why the presence of the bedsore was reported."

It is a question whether the injury in this case should properly be regarded as a bedsore. In the view of the matron, "it was not a bedsore, it was not a bottle burn, it was not a scald, but I thought a nurse had allowed a blanket to get too hot." The lesion was present twelve days before the child's discharge, and the child was under treatment in the casualty department, partly at least for this injury, for fourteen days after it had left the hospital.

In our judgment the matron failed in her duty in not seeing that this "graze" was reported to Dr. Macqueen, though she herself knew of it from the sister in the children's ward, nor can we accept her excuse as valid that she allowed the sore not to be reported because she felt that Dr. Macqueen was on bad terms with sister. As this inquiry has shown, there were very cogent reasons why the residents should not at that time approve of the management of the children's ward.

VIII.

### Treatment of Surgical Shock.

Particulars of this case are given in The Hospital (p. 646b) as under:—

4. "A patient who had had a prolonged abdominal operation was taken to a cold ward where the fire had been newly lit, where there was only one hot-water bottle in the bed; and was left, still under the influence of the anæsthetic, upon the trolley, with no one to look after her. The patient died the same afternoon."

From the evidence it appears that the patient's name was Frances T., aet. fifty-five, originally under the care of Dr. Evelyn, but afterwards transferred to Mr. Jalland for operation as a case of acute intestinal obstruction.

According to Dr. Macqueen's evidence, the operator was Dr. Charles, at that time house surgeon, and Dr. Macqueen alleges that Mr. Jalland did not see the case, and was never present.

We are unable to accept Dr. Macqueen's evidence on this head. The operation admittedly involved laparotomy, followed by the removal of fourteen inches of strangulated intestine. It is most unlikely that such an operation should have been permitted to a house surgeon alone, and it is unintelligible how a house surgeon could have performed it single-handed, his colleague being meanwhile engaged in giving the anæsthetic.

The facts appear to be accurately stated by Mr. Jalland, whose evidence is to the effect that he himself made the abdominal incision, and finding the condition of things above described, was assisted throughout by Dr. Charles, the operation requiring, as he remarks, two sets of hands.

Mr. Jalland's recollection is corroborated by the evidence of the theatre sister and a nurse, both present at the operation. On the other hand, the entry in the operation-book ascribes the operation to Dr. Charles, the house surgeon, and Dr. Evelyn, though present in the theatre, cannot "honestly say that he remembers seeing anything of the operation at all." There is, however, no great difficulty in understanding how the house surgeon might have been entered as the operator, since he was admittedly sharing the work with Mr. Jalland, nor how the entry might remain unobserved and unchallenged by either party until Mr. Jalland saw it a few weeks before the inquiry.

The patient after operation was taken to Ward No. 1, which, having been emptied for periodical cleaning, had just been got ready for the reception of patients.

We had it in evidence that there had been a fire in the ward on the previous day, and that there was a fire when the patient was moved down from the operating theatre. As to the hot-water bottles, it would appear that, as two only were in the bed the patient was to occupy, she was placed, still on the theatre trolley, in front of the ward fire for some little time, whilst more hot-water bottles were being got ready. There is nothing to countenance the suggestion that any harm was done to the patient either by the temperature of the ward or the want of hot-water bottles.

The statement that the patient was left upon the trolley with no one to look after her appears to be inaccurate. The patient was received by Sister C. from the operating theatre, and the sister's evidence is that she never left her until the "special" nurse took charge of her, and it may be observed that Dr. Macqueen ultimately limited the time during which the patient was left to "not more than five minutes."

We report that in our opinion this complaint is unsubstantiated.

IX.

### Washing of a Patient Suffering from Shock.

5. "A patient admitted after an accident (suffering from slight concussion and shock, and who had an exceedingly bad pulse—not in any fit condition to be moved or washed) was found in the process of being washed ten minutes after admission." (The Hospital, p. 646b.)

The patient concerned, Alfred F., aet. 34, was admitted on December 24, 1912, and discharged on January 2, 1913.

He had sustained a blow from a crane, which struck him on the head, and he was also thought to have received abdominal injuries. Moreover, according to Dr. Shepherd's evidence, "he had a form of heart disease, was only semi-conscious," and "in a very bad and shocked condition."

Having been seen by Dr. Shepherd in the casualty department, he was sent immediately to the ward, "with orders that he should be put to bed and left perfectly quiet." There is, however, some doubt whether these orders were ever given, for, since the inquiry we have received a signed statement by Mr. J. G. Carlile, carriage trimmer, who accompanied the patient to the hospital, and was in the casualty room the whole of the time Dr. Shepherd was there. He states that the doctor gave no specific directions respecting the treatment of the patient. However this may be, the patient was taken to the ward by the hospital porter and Mr. Carlile only, unaccompanied by any nurse, and the orders, if given, could not have reached either the ward sister or nurse.

Dr. Shepherd goes on to say that ten minutes later, whilst on his way to his own ward he passed the door of the ward into which the patient was being admitted, went in, found screens round the bed, and patient lying on the top of the bed in the process of being washed. One nurse "washing him down, whilst the other was drying him," and fully half the body uncovered. Here again Dr. Shepherd's account is directly disputed by the sister, who takes the point that ten minutes is too short a time to undress and prepare a patient for washing, and that as a matter of fact Dr. Shepherd did not arrive until at least half an hour, fixing that as the minimum that it would have taken to have got the man undressed and prepared for bed. The nurse's evidence coincides with the sister's as to the length of time which elapsed before Dr. Shepherd came into the ward, but she asserts that when Dr. Shepherd actually saw the patient, so far was he from being uncovered and in process of being washed, that he had several blankets and hot bottles round him, and "we were just about to start." Mr. Carlile states that he assisted the ward nurse "to put patient to bed, undressing him, etc.," and that this process occupied "from twenty minutes to half an hour," after which he went down to the casualty department, and still found Dr. Shepherd there.

With regard to the gravity of his condition, neither the sister nor the nurse regarded the patient as suffering from severe shock or heart failure, whilst Mr. Carlile says that he was not unconscious, "as he cried and expressed a wish to be at home." Moreover, in the absence of any orders reaching them from Dr. Shepherd, it was reasonable that they should exercise their discretion as to how to wash the patient, nor did any harm come of it. This complaint, in our judgment, lacks confirmation and substance.

### X.

### The Slipper Case.

6. "A case taken to the secretary's office without slippers on her feet." (THE HOSPITAL, p. 646B.)

This case was treated by the residents' counsel as a specific instance of the allegation made in THE HOSPITAL, p. 648B-6. "In several instances responsibility which ought to rest with a sister (in cases of mistakes and otherwise) was attempted to be put upon nurses."

This allegation concerns the treatment experienced by a patient, Mrs. Fawcett, on the morning of her discharge from the hospital. It appears that she walked to the secretary's office to sign the discharge papers with stockings, but not slippers on her feet.

The circumstances are very fully disclosed in the Minutes of the House Committee under date October 12, 1912, which were read at our inquiry, and need not here be set out.

The sister affirmed that she told the nurse to get the slippers, and the nurse was censured for not ascertaining that they were on her feet before the patient left the ward. The House Committee attributed the blame to the nurse, the residents would put the blame on the sister, and, from a letter which appeared in the *Yorkshire Herald* of April 26, 1913, it appears that Mrs. Fawcett's sympathies are with the nurse, whom she regards as the "most humane and thorough nurse that she came in contact with during her stay at the hospital."

It is clear that the House Committee took great pains to investigate all the circumstances of this case, and, though their findings were not acceptable to the residents, we cannot conclude that in this instance "responsibility which ought to rest with a sister was attempted to be put upon nurses."

### XI.

### Alleged Inefficiency of a "Special" Nurse.

The allegation is thus set out in THE HOSPITAL, p. 646B:

7. A case of diphtheria on which tracheotomy had been performed and which required special nursing. A nurse was sent as "special" who had never seen a tracheotomy before, while one who had had a fever training and nursed many similar cases was going about that night doing little or nothing. Result: Membrane was allowed to accumulate in the tube, the tube was coughed out of the wound, and the nurse was unable to replace it. We were urgently called to the case several times before this, and in the end a second tracheotomy (low) had to be performed.

This was the case of a child who was brought to the casualty department suffering from laryngeal diphtheria in a condition of such distress that Dr. Shepherd had at once to perform tracheotomy. The patient was struggling considerably and no anæsthetic was used. We learn, however, that there was no great difficulty in inserting the tube, and that the tape, which secured it in position, was fastened by the operator. The child was taken to the ward, accompanied by the resident, and received by Sister W., and, according to her evidence, Dr. Shepherd "had not gone five minutes when the tube came out." The sister is clear that the child did not itself pull the tube out, and it could, therefore, only have come out either because it was never properly in the trachea or was not properly tied in, and for both these things Dr. Shepherd was responsible. With some difficulty the sister replaced the tube without any assistance from the residents, but later on in the evening the tube again came out—the child seemed to cough it out, says the nurse—and was then replaced by Dr. Macqueen by the aid of dilating forceps. Later still, a second operation was performed (low tracheotomy) by Dr. Macqueen, and after this no further difficulties arose, the child ultimately making a good recovery, and the "special" nurse being complimented by Dr. Shepherd on the result.

After a careful study of the case we have reached the conclusion that there is no evidence that the "special" nurse was inefficient, and, moreover, we find that she was adequately supervised by the day and night sister, and that she was not responsible for "membrane being allowed to accumulate in the tube," if, indeed, it did so accumulate, or for the tube being coughed out, or for the second operation. The matron quite candidly explained that she chose the particular nurse as "special" for the case because she was a senior nurse, and she wished her to have experience in tracheotomy before finishing her training.

Under these circumstances, it becomes unnecessary to consider whether the matron ought to have sent another nurse to the case, particularly as we learn that the nurse concerned, so far from "going about that night doing little or nothing," was engaged as night nurse in a ward of more than twenty beds.

In justice to the residents, we should perhaps add that we believe the night sister and "special" nurse to be in error in supposing that between the first and second operation the incision was lengthened by a scalpel, when, as above mentioned, the tube was replaced by the aid of dilating forceps; nor do we attribute any importance to the night sister's evidence with regard to the cotton-wool or the part of the trachea, which, she says, the residents thought had been coughed out of the wound.

XII.

### Alleged Incompetence of a Ward Nurse.

This charge appears in THE HOSPITAL, p. 647A, as follows:—

8. A nurse who had only had four months' training was left in charge of the children's ward at night.

It appears that on January 13, 1913, for the half of one night and the whole of the next night the children's ward, with twenty-four beds, and occupied by fourteen children the first night and several more on the second, was placed in the charge of a probationer who had had between three and four months' training. Of the total number of patients one child was convalescent from typhoid fever, one had empyema following pneumonia, where, with a normal temperature, the pulse and respiration were rapid, one had St. Vitus' dance mildly, and one was a bottle-fed baby—according to Dr. Macqueen's recollection, a case of osteomyelitis. The other children were less seriously ill. Since the probationer had already been some time in the ward, knew every case, and "was particularly capable with them," we do not think that it was improper to leave her in charge for a night and a half on an emergency arising through the sudden indisposition of the regular night nurse, and this course appears to us to be preferable to the alternative suggested by the residents that the matron should have put on duty a nurse of longer experience who actually on that night was engaged in the isolation ward nursing a child which Dr. Shepherd describes as a case of "scarlet with an empyema—which might have been scarlet."

In our judgment this allegation lacks substance.

XIII.

### Delay in Taking Cases to the Operating Theatre

From THE HOSPITAL, p. 647A:—

9. Delay (sometimes as much as two hours) in taking a patient to the theatre after instructions had been given for the case to be in readiness at a certain time.

The residents' counsel explained that there was a printer's error here. The allegation was not intended to be that there was a delay of two hours, but that delay occurred in bringing patients to the operating theatre, even when there had been two hours' warning that the patient would be required at a certain time.

Dr. Macqueen stated that the complaint referred particularly to emergency cases, and he quoted, as a specific instance, a patient upon whom Mr. Gostling was about to operate, and in whose case there was a delay of twenty-five minutes.

Mr. Gostling, questioned as to whether it was about twenty-five minutes, said, "Yes, I cannot remember exactly the time, but I think I was waiting in the theatre; and when you are waiting it seems a very long time; and if it was twenty-five minutes it would seem a long time."

It was further given in evidence at an inquiry before a sub-committee of the Medical Board (Notebook 2, p. 87) by a ward sister that "delay in getting cases to the theatre is apt to occur, owing to shortness of staff, when there is a succession of cases to go to the theatre."

We do not know what view was taken by the sub-committee of this allegation, but in some degree it supports the complaint of the residents that delay might occur even when notice had been given as to the time of operation. That a delay of something under half-an-hour actually occurred in one emergency case is confirmed by Mr. Gostling's evidence.

XIV.

### Laxity in Carrying out Orders.

From THE HOSPITAL, p. 647A:—

10. General laxity in carrying out orders.

Examples quoted by Dr. Shepherd were VI. and IX., already dealt with, and instances in the children's ward in the days of the late Sister Victoria, which may be regarded as highly probable.

He also made a general statement as regards another ward that, having ordered purgatives for certain patients, he found on his subsequent visit that they had not been given.

Probably the sister concerned, who has now left the hospital, could have given a satisfactory explanation of some of these occasions. In her absence we cannot carry the matter further. Dr. Shepherd, questioned by Mr. Procter, admitted that these irregularities were not to his recollection reported to the honoraries in charge of the cases, and never to the matron.

XV.

### Failure to Chart Pulse and Respiration.

From THE HOSPITAL, p. 647:—

11. "Neglect to take pulse and respirations already referred to in children's ward, but also found in other wards."

The truth of this charge so far as it relates to the children's ward in the days of the late sister is admitted, and Dr. Macqueen gave instances from another ward.

Having inspected the charts of patients in that ward during the month of last December, we found that, while the temperature is always recorded, in cases where the temperature is below 99 the pulse and respirations are often not charted.

The sister of the ward admitted the facts, but explained that many of these cases were of a chronic character (*e.g.* ulcers, and senile gangrene), where it appeared to be unnecessary, and she made the point that at any rate Dr. Macqueen had never complained to her, nor had the honorary staff ever raised objections to her practice.

XVI.

### Negligence in Reporting Deaths.

From THE HOSPITAL, p. 647:—

12. "Frequent failure to report deaths to the resident in charge."

The specific instances relied upon by Dr. Shepherd are six, already considered; a child dying during the night, whose death should have been reported by the night sister before she went off duty; and a man whose death should have been reported by the day sister. Of this death Dr. Macqueen first learnt from the arrival of the friends to ask for the death certificate.

It appears to be the custom of the hospital that, when a death occurs, the resident is informed verbally by the responsible sister, or, if this is not practicable, by a slip of paper placed in his room, and at the same time notice is sent to the matron.

As regards the last case, the ward sister is unable to state whether she actually sent a nurse to notify the resident of the death, but she is quite clear that Dr. Macqueen came to the ward and ordered strychnine for the patient "very near the end," and it is probable, therefore, that she did nothing further when the man was actually dead, as he had been seen so shortly before death.

The second case was a child three weeks old, who died at 7.20 a.m., and whose death should have been reported to Dr. Shepherd; and, in spite of the night sister's assertion that she would have been "most particular," and would either have gone to his bedroom before breakfast or would have left a note upon the resident's dining-table, we are not satisfied that as a fact either of these things was done.

### XVII.

### Absence of the Day Staff from the Wards.

From The Hospital, p. 647a :—

13. "It was a frequent occurrence to pay a ward visit and never see a sister or nurse until the visit was almost finished—sometimes not at all."

More circumstantially it was complained that the residents might be in a ward from five to ten minutes without seeing a nurse, and Dr. Macqueen asserts that sometimes the delay was "as much as twenty-five minutes."

The explanation, as regards, at all events, the shorter interval, appears to be that on the surgical side it is customary for the sister and all the nurses to have a break of a quarter of an hour for an early lunch about half-past nine or a quarter to ten o'clock, during which interval they retire from the ward to the day-room, which is so close that in an ordinary way they can hear the doctor when he comes up.

During the time the nursing staff is absent there is a ward maid on duty in the ward, but it may happen that she is engaged in the annexes, and so is not there to tell the sister of the arrival of the resident, and the sister herself may not have heard his footsteps on the stairs.

It was stated to us by the matron that it was only when there was no serious case, and practically on the surgical side only, that the nursing staff was ever absent in a body from the ward. We think, however, that in the interests of the patients no less than of the residents this custom might well be entirely discontinued.

### XVIII.

### Lack of Ward Etiquette.

From The Hospital, p. 647a :—

14. "There was a general lack of ward etiquette."

It was explained that this referred more particularly to the facts just reported on, as to the absence of the nursing staff during the visits of the residents to the wards, if these visits should occur during the quarter of an hour occupied by early morning lunch. Dr. Shepherd also complained that, when a resident had done anything in the ward which required him to wash his hands afterwards, he was frequently allowed to pour out the water for himself. The further point raised, but not pursued, was that a certain sister had told a resident that his "behaviour was stupid."

It was admitted by Dr. Macqueen that it could hardly be made a rule of the hospital that nurses should pour out water for the residents, and that much must depend in these matters upon the maintenance of friendly as distinct from purely official relations.

It seemed unnecessary to pursue the inquiry further.

### XIX.

### Alleged Inadequacy of the Nursing Staff.

From The Hospital, p. 647a :—

16. "Frequently external nurses had to be obtained, as the hospital staff was so inadequate."

Obviously it is a merit that external nurses should be obtained, and the allegation repeats what is said under III. : that the regular nursing staff of the hospital was inadequate in number, and particularly, in the residents' opinion, as regards the theatre staff and the supply of "special" nurses for operation and other serious cases.

On this point we had before us a report by Drs. Macdonald and Gayner, dated February 6, 1913, in which they come to the conclusion that there is need for an increase of the nursing staff in the eye ward, and that there should be an extra nurse in the operating theatre at night. They further urge extreme care in ordering "specials," and recommend the avoidance of operations between twelve noon and two o'clock, except in cases of emergency.

Dr. Macdonald told us that "if we had not a treasurer, and had an unlimited amount of funds, we might have asked for very much more," and the allegations of the residents, therefore, are practically admitted by the staff, even though it is stated that the number of nurses to patients (1 to 3.6) at the York County Hospital is somewhere about the average as given in Sir Henry Burdett's Hospitals and Charities Annual.

### XX.

### Food and Personal Laundry.

From The Hospital, p. 647a :—

"The food was always extremely monotonous, on many occasions unpalatable, and several times uneatable."

No definite occasions were cited to us to substantiate these complaints, and Dr. Charles asserts that during the three or four months that his residentship coincided with Dr. Macqueen's "the food to my mind was splendid," and that, having had experience of the dietaries of various hospitals, he finds that the York County Hospital "beats all of them."

We did not further investigate the subject, nor yet the grumbles as to the personal laundry of the residents.

### XXI.

### The Accommodation of the Residents.

From The Hospital, p. 647a :—

"Regarding our personal treatment in the said hospital, our living apartments were, in our opinion, in an extremely unsatisfactory condition."

We took an opportunity of inspecting the quarters of the residents, and in our opinion they compare very favourably with what is found in English Universities and in other hospitals. They consist of a large dining-room—a portion of which is partitioned off as a cloak-room for the honorary staff—a common sitting-room, and separate bedrooms.

Except that the wallpapers must be somewhat shabbier, the rooms are in the same state as when the residents left last January. Dr. Shepherd informed us that the paper in his bedroom having become loose from damp, he had ensured the re-papering of the apartment by the simple expedient of pulling off the flapping piece, and had thus put his room in such a condition that "it had to be done." At our inspection, no trace of damp remained.

We cannot but think that this complaint is of a very unsubstantial character.

### XXII.

### Retirement of Residents.

On December 10, 1912, the House Committee, according to an extract from their minutes, granted leave of absence for ten days to the house surgeon and house physician, to run consecutively. In subsequently cancelling their holidays the House Committee are accused by the residents of having adopted a "very dog-in-the-manger attitude." In view, however, of what happened within a week of the meeting, we cannot think that their "attitude" was unreasonable, for on December 24 "the Committee were informed that on Sunday, the 15th inst., the resident officers had broken their tea service, as they contended it was unfit for use, owing to its cracked condition. Broken pieces were examined, and from what could be seen the Committee were satisfied that the action was not justified."

"Dr. Macqueen, the house surgeon, and Dr. Shepherd, the house physician, were interviewed separately, and were asked by the Chairman if they had any explanation to offer, and the reply was that they had complained, but no notice had been taken. It was pointed out that before taking such drastic steps they should have brought the matter to the notice of the Committee. However, both officers maintained they were in the right, and intimated that they would be inclined to do the same thing again in similar circumstances."

After a short discussion it was resolved :—

"That the house surgeon and the house physician be given one month's notice to terminate their engagements, and that the leave of absence granted at the last meeting be withdrawn."

This resolution was passed after Dr. Macqueen and Dr. Shepherd had left the Board Room.

Mr. Neden explains that he did not at once write the residents an official letter giving them notice to terminate their engagement, because he desired still to afford them an opportunity of apologising. This, however, they declined to do, and on December 27 they sent a letter to the Chairman of the House Committee, which they term a "letter of resignation," but which is actually a conditional resignation thus phrased :—

"Unless radical changes along these suggested lines are promptly made we, the undersigned, cannot see otherwise than to tender you our resignations separately and conjointly."

The formal letter from the Committee was not sent until December 31, when a special meeting of the House Committee reaffirmed the previous resolution of the 24th.

In reply to this communication the residents made the following points :—

(1) That they had already resigned by their letter of December 27.

(2) That their dismissal by the House Committee was not in accordance with the rules under which they were appointed.

As regards the first point, it may be objected that their resignation was conditional only, but as regards the second point their contention appears to us to be justified, since by the regulations of the hospital the appointment and dismissal of residents is made by the House Committee and the Medical Board sitting jointly, and the members of the Medical Board had not been summoned to take part in the resolutions either of the 24th or of the 31st of December, nor are the proceedings regularised even though the representatives of the Medical Board upon the House Committee were present and assenting, and though at a later meeting the Medical Board expressed its concurrence in what the House Committee had done.

An extract from the special meeting of the House Committee and Medical Board, held January 21, 1913, informs us that the question of the termination of the period of office of the resident officers was considered, and that "the officers concerned had expressed their desire to leave on the 23rd instant, and it was decided to agree to this." In other words, they retired, and were not dismissed.

### XXIII.

### The Question of Testimonials.

From THE HOSPITAL, p. 648A :—

We terminated our engagements at York County Hospital on January 23, 1913.

On the day prior to the appointment of new residents one of us asked a member of the honorary staff for a testimonial. He said he would consider it. On the same evening a second member was also asked for a testimonial. He said it had been suggested that we should get testimonials if we would promise not to say anything to the candidates who were coming the following day regarding the condition of affairs existent in the place. On the following morning the member of the honorary staff first referred to came to the hospital and informed one of us that he had considered the matter, and that he would grant a very good testimonial if an undertaking was given him that we should say nothing about the place to the candidates who were coming that day. This appeared to us to show that these testimonials were to be granted in the light of "hush-money," and they were refused. Both men admitted that our work, both medically and surgically, had been extremely satisfactory.

The suggestion that the residents should receive testimonials only upon certain conditions appears to have come from Mr. Jalland, who in his evidence explains that the conditions contemplated were that they would not upset the men who were to follow them as residents by telling them of their complaints about "food and things of that kind."

Acting upon Mr. Jalland's suggestion, and feeling also that any testimonial he gives implies an assertion as to the "loyalty of the person who receives it from him," Dr. Macdonald desired as a condition of Dr. Macqueen's testimonial that he should agree "not to say what he said here to the candidates," and "not to prejudice them against the place." He added that what he had in his mind was that the outgoing residents should not "go out of their way to prejudice the new men against the place" if there were anything unsatisfactory in their relations with the matron, or in regard to food, etc., all of which things the new men might be left to find out for themselves, but that he had not in his mind the graver allegations which have been made in the course of this inquiry.

Dr. Macqueen found himself unable to accept these conditions, and received no testimonial.

Dr. Shepherd, however, had a testimonial sent him by Dr. Macdonald on the ground that he was not primarily responsible for the trouble, but that he was being carried away by Dr. Macqueen, and he was also understood by Dr. Macdonald, though any such undertaking is denied by him, to have promised not to prejudice the on-coming residents against the place.

As regards Mr. Gostling, he also took the line that he would give Dr. Macqueen a testimonial if he would not "crab the place to the new men who were coming," explaining that he meant "chiefly about the food and their accommodation."

Apparently, without any undertaking to this effect from Dr. Macqueen, he promised him a testimonial, but out of sheer forgetfulness omitted to carry out the promise.

We cannot but come to the conclusion that the action of Dr. Macdonald and Mr. Gostling was ill-advised under the circumstances, and was likely to lead the residents to think that their testimonials were, as they say, to be granted in the light of "hush-money," and therefore rightly to be refused.

XXIV.

### The Treatment of Sick Nurses.

The specific incidents relied on to support the allegation of the unsatisfactory treatment of sick nurses were as follows :—

(*a*) A case of diphtheria, where the patient had been in the nurses' home for several days prior to being seen by the resident or anti-toxin administered. (The Hospital, p. 648 a and b.)

It appears that this case was reported by the matron to the House Committee on October 22 as follows :—

"Nurse D., who was stated at the last meeting—a fortnight previously—to be suffering from sore throat, has developed diphtheria, and is going on satisfactorily."

The assertion that she had been in the nurses' home for several days prior to the administration of anti-toxin is directly negatived by the evidence of Dr. Evelyn, and by the entry on the temperature chart of the case produced for our inspection.

The nurse, who complained of a sore throat, took to her bed on October 2. She was seen by Dr. Evelyn the next morning, when, finding her to have "a rather suspicious throat," without waiting for the result of a bacteriological examination, he ordered Dr. Shepherd, the house physician, at once to inject a dose of 4,000 units of anti-toxic serum.

The temperature chart, with the date of the administration of anti-toxin thus recorded, was presented to Dr. Shepherd, and was not disputed, and a letter was read from the nurse to the matron that she was reported sick on "Tuesday, October 1, or on Wednesday, the 2nd, and was having anti-toxin the same evening."

The charge that the nurse had been in the home several days prior to being seen by the resident or anti-toxin administered appears to us to be clearly disproved.

(*b*) "A night nurse, who was subject to heart attacks, and who had complained during the day, was found by us, on duty at night, cyanosed and gasping for breath, in no fit condition to be even sitting up. Although ordered immediately off duty, she had to remain two hours longer until a day nurse had had a sufficiency of sleep to take her place." (The Hospital, p. 648b.)

The nurse concerned appeared before us, and denied that she was at all in the condition represented by the residents. "I just had a pain in my side."

The certificate of her own doctor when she entered the hospital on April 18, 1910, described her heart and other organs as "good"; she was passed as sound by Dr. Evelyn, physician to the nursing staff, on August 22, 1910, and when "critically" examined by him on the third day after she had this "cyanosed attack in the ward," he found "nothing wrong with her heart, no disability of any kind." By his advice she returned to duty, and "she has remained on duty ever since."

We report that this charge is unsubstantiated.

(*c*) "A case of sinus suppuration and threatened frontal trouble was left in her bedroom in the month of December without a fire, and seemingly no thought of it until ordered." (The Hospital, p. 648b.)

This was the case of a nurse who was under treatment as an out-patient by Dr. Macdonald for nasal trouble, and, as she did not improve, Dr. Macdonald asked Dr. Shepherd to see she was put to bed in the nurses' home. The same day she was visited in the home by Dr. Shepherd, who found no fire in the room, and on the next day, when seen by Dr. Macdonald, he ordered a fire, not, as he explains, for any other reason than that he might induce sweating in the patient, whose case he regarded as of quite trivial importance.

As the matron explained, fires were not as a rule lighted in nurses' bedrooms, and usually, when the condition of a sick nurse is such as to require a fire, she is transferred as a patient to a ward.

This complaint is obviously of a trivial character.

(*d*) "A case of severe abdominal colic first felt by the nurse on a Sunday. She reported herself to the sister on Monday; no notice was taken. She reported herself again, to the matron, on Wednesday, and was not seen by a doctor until Thursday. At table this nurse had been noticed to have taken little or no food, and had asked one evening to be excused from attending the meal." (The Hospital, p. 648b.)

This also is a complaint of little substance. The nurse concerned was seized with abdominal pain on a Sunday, and it was attributed to a natural cause. On the Monday she spoke to her ward sister about it, who suggested castor oil. On Tuesday morning, when she made her round, the matron learnt of her indisposition from the ward sister, and arranged that she should spend that afternoon as a half-day off duty, resting in the home. On the Wednesday morning, she found that the nurse, instead of doing so, had gone into the town visiting, or, as the sister puts it, "although the weather was very cold, she went out to tea." On Wednesday she said she was feeling better, and went on duty, but on Wednesday night at supper-time she asked to be excused, and the matron then ordered her to bed "and not to get up again next morning." She was seen on the Thursday by Dr. Evelyn, and daily by him for four days in succession; Mr. Gostling was called into consultation; a surgeon in Newcastle, under whose care she had been, was written to; and finally she was sent away on holiday.

We have thus reported upon the specific instances of alleged neglect in the treatment of the nursing staff when sick, and we find that they are not sustained by the evidence brought before us. On the other hand, it can hardly be doubted that the known difficulties of attending to them in the home, particularly at night, and the want of a sick-room for those who, though not fit to go on duty, are yet not ill enough to be warded, must tend to "the general hesitancy" alleged by the residents "on the part of the nurses themselves to report if they were ill." As a matter of fact, we find from notes of evidence on the nursing question given before the sub-committee of the Medical Board (January 19 to February 5, 1913), vol. I., p. 59, that the matron "considers provision of sick-room for nurses urgently required, as there is no staff on the spot at all hours in the home, and the strain on the assistant matron looking after nurses in the home is considerable. Also when it is wished to transfer a nurse from the home to a ward, there is not always a bed available."

This opinion of the matron is endorsed by the report of the sub-committee (Dr. Macdonald and Mr. Gayner) above mentioned. On page 6 of that report it is stated that "there is a pressing need for further accommodation for sick nurses. When they can be 'warded' the existing arrangements are satisfactory; and the same may perhaps be said in regard to cases which are slight or of brief duration, when the sick nurses can well stop in their own rooms. But it is clear there is much need for a nurses' sick-room so placed that it can be conveniently worked in connection with a ward."

"We recommend for a period, as experiment, the earmarking of the side ward in 'Victoria' as a nurses' sick-room," and as the gist of their recommendations to the House Committee, the sub-committee "would respectfully suggest the importance of the provision of a sick-room for nurses."

XXV.

### Alleged Misconduct of a Sister.

5. "In one of the wards a sister, losing control of herself, threw a book at a nurse in front of the patients." (THE HOSPITAL, p. 648B.)

The nurse concerned appeared before us and stated that the sister was annoyed with her for giving her an incorrect record of a patient's temperature, and that she slapped her hand with the temperature book, which dropped to the floor. The nurse rightly felt aggrieved that the sister should behave as she did, particularly in the presence of the patients. Such conduct, if proved against the sister, would deserve severe censure, but having learnt that she had left the hospital, and was "not available," we were unable further to carry our investigation.

XXVI.

### Alleged Unhappiness of the Nursing Staff.

"A glance over but a few of the facts placed before you must be more than sufficient to convince you of the unhappy position in which the nurses found themselves, self-condemned to a so-called training of three years. Is it any wonder, therefore, that some of them going off on holiday forget to return—one, we believe, even paying (while on holiday) one month's salary in lieu of notice?" (THE HOSPITAL, p. 648B.)

We took no evidence on this head from the residents, and we do not certainly know what instances they had in mind. The only case known to the hospital authorities as countenancing the allegation is that of a Nurse B., who, whilst on leave, wrote to the matron on November 18, 1912, to say "I am not returning to the hospital. I hope it will not cause any inconvenience." To this the matron replied that, as she was in receipt of a salary, it would be necessary for her to give one month's notice. On the 23rd, the nurse writes that she had no idea that she should have to give a month's notice, and that that being so, she will return, but desires to postpone the date until the early part of January. On the 29th, she pays a month's salary in lieu of notice, and adds: "I may as well let you know now as later that I am going to be married early in the spring, and that I do not really wish to return."

This correspondence seems to indicate that it was the happy rather than the unhappy position in which this nurse found herself which decided her to relinquish her training.

### Conclusion.

9. We thus, therefore, conclude our report on the result of our investigations into the charges made against the York County Hospital in THE HOSPITAL newspaper, nor is it any part of our reference to make suggestions for the avoidance in future of such regrettable incidents as the present inquiry has disclosed.

The final paragraph, however, of the residents' communication to THE HOSPITAL alleges that "neither the Medical Board nor the House Committee of York County Hospital would take notice of our reports or even attempt to aid us in remedying the existent and so apparent evils."

This statement is far from accurate, as the evidence has shown, but it not unnaturally comes from the irritation engendered in their minds by the ignorance in which they were kept as to what was being done by those in authority, and what we would venture with all respect to suggest to the Governors is that, as is the practice in some other hospitals, the residents should have the privilege of regularly appearing before the House Committee with their journals, in which they should record any statistical or professional details required of them, and in particular should be at liberty to call attention to grievances, and to make suggestions for improvements in the administration of the institution. They are, by the necessities of their work, brought into personal touch with the honorary staff, and it is to our mind scarcely less important that they, and no less the matron, should have ready access to the chairman and members of the House Committee, with whom rests the ultimate responsibility of administration. The residents will usually be young men, with the natural impatience of youth; they will entertain a high opinion of the importance of the functions they discharge in the hospital; they will be zealous for new things; the more desirable is it that they should be given full opportunities to advocate changes, to ventilate opinions, and to bring their suggestions to the notice of the House Committee.

We feel convinced that, had such facilities been afforded to the late residents, whatever of ultimate good may come of our investigations might equally well have been attained without the trouble, annoyance, and waste of time better spent, which this inquiry has entailed upon all connected with the York County Hospital.

E. COOPER PERRY.
BASIL WATSON.

May 29, 1913.

